# Editorial: Engineered immune cells in cancer immunotherapy (EICCI)

**DOI:** 10.3389/fimmu.2022.1119363

**Published:** 2022-12-20

**Authors:** Francisco Martin, Axel Schambach, Cristina Maccalli

**Affiliations:** ^1^ Gene and Cell Therapy group, Departamento de Bioquimica y Biología molecular 3 e inmunología, Universidad de Granada, Center Pfizer-University of Granada-Junta de Andalucía Centre for Genomics and Oncological Research, Granada-Junta de Andalucía Centre for Genomics and Oncological Research (GENYO), Granada, Spain; ^2^ Institute of Experimental Hematology, Hannover Medical School, Hannover, Germany; ^3^ Division of Hematology/Oncology, Boston Children’s Hospital, Harvard Medical School, Boston, MA, United States; ^4^ Research Department, Sidra Medicine, Doha, Qatar; ^5^ College of Health and Life Science, Hamad bin Khalifa University, Doha, Qatar

**Keywords:** adoptive cell theraphy, CAR-T cells, TCR-engineered T cells, tumor antigens, mechanisms of resistance

Adoptive Cell Therapy (ACT) to treat cancer represents a rapidly evolving field. New approaches for the genetic engineering of immune effector cells with either a T cell receptor (TCR) or a chimeric antigen receptor (CAR) (**Figure 1** *Panels A and B, respectively*) have led to the increase of the clinical efficacy, the reduction or better control of toxicities and the expansion of the indications of these therapies. The Research Topic “Engineered Immune Cells in Cancer Immunotherapy (EICCI)” represents the venue for collecting studies, new evidence, advances in the technologies and the greatest knowledge for the translational application on the topic of cellular therapy for cancer. The great success of this Research Topic with the publication of total 46 articles, including 18 original articles, 20 reviews, 5 mini reviews, 2 case reports and 1 methodology manuscript, and the contributions of 360 authors, testify to the huge interest of the scientific community in this field and the numerous advancements.

**Figure 1:**
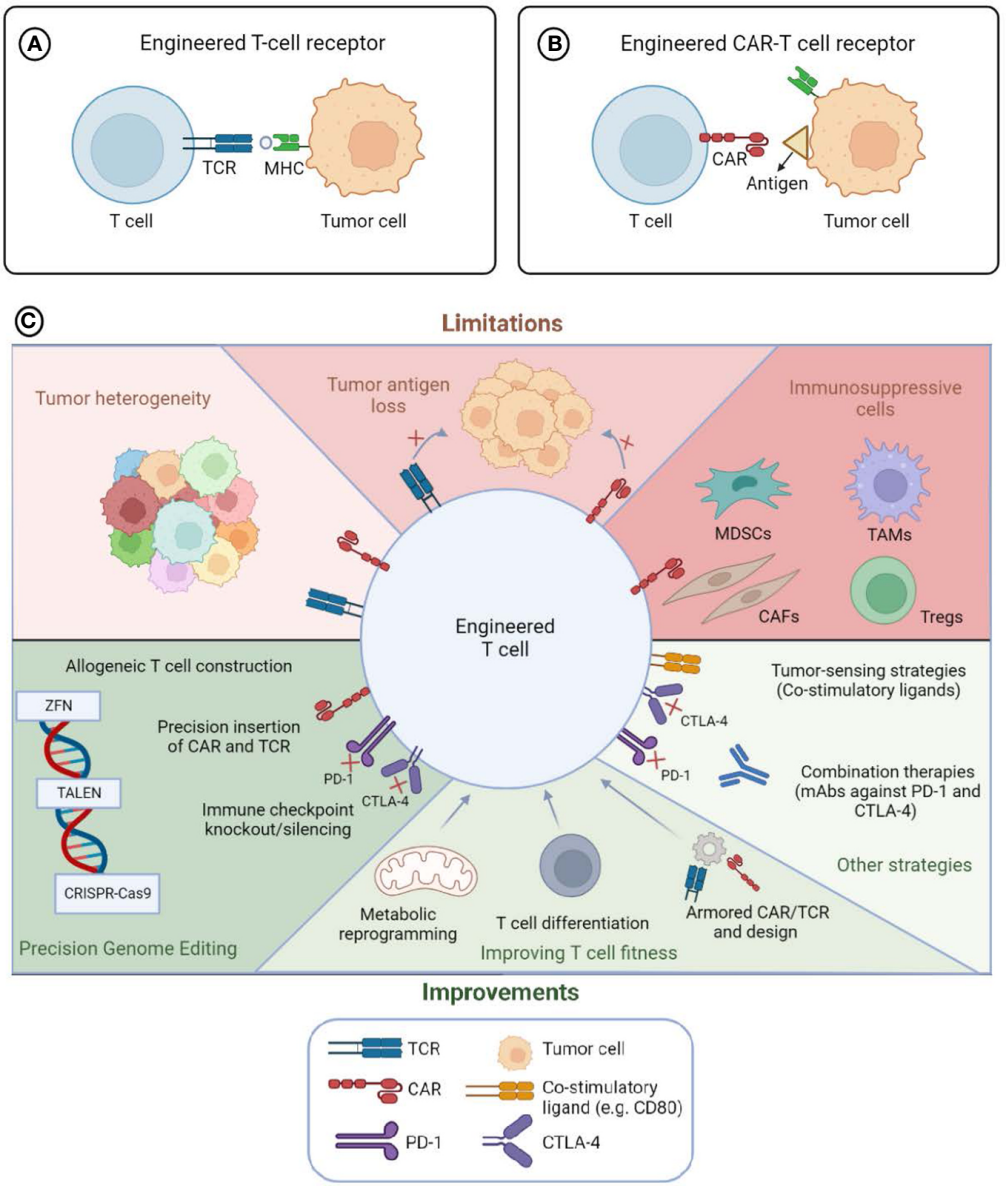
Engineered T cells to target tumor cells: limitations and advances. T lymphocytes engineered with T cell Receptor (TCR) (Panel **A**) or Chimeric Antigen Receptor (CAR) (Panel **B**) to redirect the immune cells toward tumor cells. The survival, proliferation and anti-tumor functions of engineered immune cells (Panel **C**) can be influenced by intrinsic factor of tumor cells, such as cell heterogeneity, antigen loss, or the immunosuppressive tumor microenvironment (TME) (Top part of Panel **C** entitled limitations). The administration of immune checkpoint blockade agents or co-stimulatory molecules in combination with engineered lymphocytes can modify the tumor milieu and implement the anti-tumor functions of the cellular products. Moreover, the modification of the fitness of the lymphocytes through actions that can change either the metabolomic profile, the differentiation status or the cytokine or immune checkpoint expression can lead to potentiate the functions of the immune cells (bottom part of Panel **C** entitled improvements). Precision genome editing (through the usage of either Zinc finger nuclease, ZFN, Transcription activator-like effector nuclease, TALEN or clustered regularly interspaced short palindromic repeats (CRISPR)/CRISPR associated protein 9 (CAS9)) can increase the expression of the antigen-specific receptors, modify the fitness of the immune cells and/or implement the safety of the cell-therapy product. MDSCs, Myeloid-derived suppressor cells; TAMs, Tumor-associated macrophages; CAFs, Cancer-associated fibroblasts; Tregs, regulatory T cells. Created with BioRender.com

The Research Topic is built on the outcomes of the 1^st^ International Workshop on Engineered Immune Cells in Cancer Immunotherapy (EICCI), held at Sidra Medicine in Doha, Qatar, on 15th-16th February 2019. Worldwide experts in the field of cell therapy gathered in Doha to discuss progress and challenges in the field. The proceedings from the workshop by Guerrouahen et al. summarized the presentations from speakers from both academia and industry on pre-clinical and clinical development of genetic engineering ACT (GE-ACT), genetically modified immune cells, organizational needs and hurdles for the clinical grade manufacturing and the clinical application of these biological drugs.

11 years have passed since the first evidences that CAR-T cell therapy could revolutionize the clinical treatment of patients with hematological malignancies, such as acute lymphocytic leukemia (ALL) and non-Hodgkin lymphomas (NHL). Following these outstanding achievements, GE-ACT underwent an unprecedented growth, as testified by the 745 clinical trials, with 641 with CAR- and 102 with TCR-based therapies, that occurred for GE-ACT until the end of 2019 [Pinte et al.]. Interestingly, most clinical trials have been developed in China (47%) and the USA (44%), followed by the EU (6%) and other countries. Until 2013, the majority (91%) of GE-ACT clinical trials were spontaneously developed in academic settings, while from 2014 on, 54% of these studies were sponsored by biotech and pharma. Nevertheless, the majority of these clinical investigations are still in the early phase of development (Phase I/II) to demonstrate the safety and efficacy of the novel product aimed at targeting a variety of tumor antigens and modified to implement their anti-tumor activities. A bibliometric analysis performed by Ou et al. confirmed the rapid progress of the field of CAR-T cells over the 2001-2021 timeline, with a total of 5981 articles reviewed and with the US being the leading country in terms of the number of publications, followed by China and Germany. Similar results were reported by Miao et al., with hot topics represented by cytokine release syndrome (CRS) associated with CAR-T cells, CD19-CAR-T cells, emerging tumor antigens to redirect the immune cells, GE-ACT for solid tumors and universal CAR-T cells.

## Regulatory/manufacturing

A comprehensive overview of the regulatory and organizational requirements of clinical centers dedicated to the administration of cell therapies to cancer patients have been provided, also introducing the novel professional profile of the CAR-T specialist deputed to the overall coordination of GE-ACT clinical unit [Gotti et al.].

An important consideration is represented by the clinical-grade manufacturing of CAR-T cells that might occur in centralized facilities vs. academic/hospital environments with associated advantages or hurdles and that might affect the accessibility as well to these peculiar therapies [Landazuri et al.]. This review also provided the difficulties posed by the COVID-19 pandemic in the preparation and administration of engineered T cells to patients.

## Clinical trials/results/reviews

Recently, two clinical grade CAR-T cell drugs have been approved for the treatment of multiple myeloma (MM), which generally relapses or is refractory to the available therapies, representing a salvage intervention for these patients. García-Guerreo et al. and Jasiński et al. provide an overview of the knowledge of CAR-T cells for MM, describing candidate antigens and multiple designs of CARs to combine costimulatory domains and/or growth factors and cytokines to modulate the strength of antigen binding of the CARs, increase the T cell survival and prevent their exhaustion. This review also suggested that the combination of CAR-T cells with other drugs currently utilized for MM, such as immunomodulatory agents, proteasome inhibitors, or antibodies, might represent the future in the therapeutic field for this type of malignancy. CD19-CAR-T cells introduced a major advance for pediatric hematological malignancies (B-acute lymphocytic leukemia; CLL) demonstrating 65-90% of complete responses across clinical trials. However, one of the major challenges following this cell therapy is the durability of the responses and the frequency of relapse. Schultz et al. addressed the limitations of the clinical outcomes associated with the CD19-CAR-T cell therapy in pediatric cancer patients highlighting the needs of identifying the predictors of responsiveness to help the stratifications of patients between long term responder and high risk of remission who might need further infusion of the engineered cells or stem cell transplantation.

## Improving CAR-T cell products: New CAR designs

The long-term clinical outcomes of these live drugs correlate with the *in vivo* persistence of T cells. Therefore, the optimization of CAR design and/or cell engineering and T cell subset selection are important considerations for the translation of cell products to clinical application.

### The cytoplasmic signaling of CARs

The cytoplasmic signaling domains of the receptor determine the persistence and survival, as well as the cytotoxic functions of T cells. The introduction of modules encoding for co-stimulatory molecules (e.g., CD28, ICOS, 4-1BB, OX40, etc.) into the design of CARs has overcome initial limitations, empowering the engineered T cells with activation, proliferative and cytotoxic properties. In this direction, Meng et al. reviewed the studies aimed at improving CAR-T cell functions by the modification of the immunoreceptor tyrosine-based activation motif (ITAM) sequence in both CD3ζand CD28ζ. The augmentation of CAR-T cell survival and persistence could also be achieved through the insertion in the modular structure of the chimeric receptor of anti-apoptotic genes (Bcl-xL), as described by Fan et al. in a model of adoptive cell therapy for solid tumor.

### Hinge domain and the transmembrane domain

Several articles in this topic analyzed how to improve CARs through the modifications of the hinge domain (HD)/ transmembrane domain (TMD). Muller et al. reported that the choice of the modular structure of the intracellular signaling can affect the functions of the engineered T cells. Biochemical differences can occur between CD28-TMD and CD8-TMD in the CAR. While CD28-TDM can have superior sensitivity to low abundant antigens on tumor cells as compared to CD8-TDM, the first TMD can augment the sensitivity to antigens expressed at low levels, leading to toxicities. CD28-TMD can form heterodimers leading to reduced CD28 expression that could lead to lower persistence and affect the differentiation of CAR-T cells. These authors showed that the extracellular spacer domain, usually the CH2-CH3 components of IgG1, of CARs is critical for redirecting T lymphocytes towards tumor cells, since this regulates the distance between effectors and target cells. CARs containing long spacer can recognize membrane proximal antigens efficiently, while membrane distal epitopes require CARs containing short spacers for the receptor engagement. Along the same line, Schafer et al. developed a novel spacer derived from Sialic acid-binding immunoglobulin-type lectins (Siglecs). The advantage of this spacer is the efficiency in binding membrane proximal antigens expressed by either liquid or solid tumor cells, such as CD20 and TSPAN8, combined with central memory CAR-T cell phenotype, high cytotoxic and low inflammatory profile. This spacer also reduces the unspecific off-target binding of the CARs.

### Binding domain

As expected, other groups have centered their attention on the CAR binding domain, as a first domain that interacts with the targeted tumor. In this direction, McComb et al. showed that CAR-T cells generated with anti-EGFR nanobodies linked with truncated CD8 hinge target selectively tumor cells over-expressing EGFR and not normal cells expressing low levels. They also showed that epitope location was critical for determining hinge-domain requirements for CARs and therefore, hinge length tuning can be used for controlling antigenic sensitivity in CARs-T cells.

## Improving CAR-T cell products: Improving manufacturing

The understanding of the mechanisms underlying the development of toxicities helped to overcome or prevent some of these effects. Few combinatorial strategies with either antagonistic antibodies, immune checkpoint blockade, Jak inhibitors, or conditional regimens have been shown either at the pre-clinical or clinical levels to overcome the inflammatory reactions associated with CAR-T cells [Safarzadeh Kozani et al.; Miao et al.. Further studies aimed at dissecting the mechanisms leading to severe side effects and to the design and manufacturing of CAR-T cells are warranted to improve the safety of these live drugs. The variability of procedures for the generation of CAR-T cells at different manufacturing sites can affect the final characteristics of the product, including the differentiation status and the survival and cytotoxic features. This field requires optimization and standardization. In this context the investigations of Arcangeli et al. and Jackson et al. analyzed the automated manufacture of CAR-T in autologous setting and could reveal important technical approaches also demonstrating that patient’s intrinsic characteristics can affect the final QC of the therapeutic cell products. Moreover, reliable tools to perform the quality controls of the cell-based product and to monitor their changes along with the manufacturing or even upon the administration into patients are desirable. One example is represented by the design and validation of immunofluorescence panels that, in a relatively simple manner, can be exploited to evaluate at the same time the expression of the CARs, the differentiation status and the cytotoxic functions of engineered T cells [Blache et al.]. These tools need to be accessible and exploitable in a large scale by laboratories at the manufacturing and/or clinical sites, without the need of complex platforms.

CAR-T cells are a gene therapy-based advanced therapy medicinal products (ATMP), and therefore, different tools and protocols to achieve these genetic modifications will render different CAR-T cell products. In this direction, several articles focus on how different gene therapy protocols can generate better products using ɣ-retroviral or lentiviral vectors. Jin et al. demonstrated that the temperature of transduction of T lymphocytes with lentiviral vectors (LVs) encoding for CARs is an important factor that can affect the differentiation and cytotoxic features of CAR-T cells. Brandt et al., Kozani et al. and Tristan-Manzano et al. [22] described the importance of using the appropriate promoters to express the CAR. Generally, strong CAR expression has been envisioned as an advantage for tumors with low antigen expression. However, if that is not the case, this strong CAR expression can lead to tonic signaling and premature exhaustion of CAR-T cells, lowering the overall anti-tumor efficacy. Another important factor is to be able to expand the CAR-T cell product without losing their most wanted properties (high % of early stage of differatiated cells). In this line, Arcangeli et al. and Jackson et al. presented optimized protocol for CAR T cell production using methods that are compatible with automated manufacturing and can generate CAR-T cells products highly enriched of stem cell memory T cells (TSCM) from patients. Although retroviral vectors are rendering impressive results so far, their production is time-consuming and costly, and other gene transfer system alternatives to achieve stable expression of CAR in T cells are being investigated. In this topic, Li et al. reported a case report of triple-hit relapse/refractory (RR) DLBCL with TP53 mutation treated with CAR-T cells generated with piggyBac transposons. This emerging non-viral methodology possesses a large cargo capacity and, more importantly, the manufacturing is simple and cost-effective. This team reported complete responses in patients, in which durability could be achieved by the combination with lenalidomide. Although further investigations are required to confirm the efficacy and safety of the combination therapy, this evidence proves the feasibility for clinical application of a different modality of gene transfer. However, this vector has been implicated in the generation of lymphoma in 2 patients from a clinical trial performed by Micklethwaite et al. (1) Alternatively, transposons based on sleeping beauty are also being investigated.

## Controlling CAR-T cell activity

Besides the excellent clinical outcome of CAR-T cells for several blood cancers, several toxicities have been observed in patients treated with these “living drugs”; some of them are life threatening if not recognized and treated at the onset of the development as discussed by Miao et al. The most common side effects observed in patients are the CRS, the immune effector cell-associated neurotoxicity syndrome (ICANs), off-target effect, anaphylaxis, B cell aplasia tumor lysis syndrome and infections. Also, to improve the efficacy of CAR-T cells against solid tumors, the secretion of potent active molecules such as IL-12 or interferons (IFNs) is required and can therefore generate even stronger adverse side effects. This toxicity also limits the efficacy, due to the impossibility to reach the appropriate concentrations in target organs. There is therefore a clear necessity to develop strategies that can control CAR-T activity in a time and spacial manner. In this direction, Brandt et al. and Tristán-Manzano et al. described the different strategies developed by different groups to control the potency and duration of the CAR-T activity.

Efforts have been devoted to regulating the expansion and the activity *in vivo* of CAR-T cells through different approaches that can be divided into those that allow the CAR-T cells to “take” decisions based on the environment (endogenous regulation) or based on the external addition of an inductor (exogenous regulation).

The idea of generating CAR-T cells that can be controlled by themselves and be active only in the presence of the tumor antigen is in the CAR-T cell DNA. However, these cells can be activated by different factors other than the targeted tumor, generating undesired side effects. This is even more problematic when designing CAR-T cells for solid tumors that require the secretion of potent immune regulators. There are multitude of approaches under study to achieve this autonomous control, which is reviewd by Brandt et al., such as the Split-CARs (the CAR binding domain and signaling domains are separated), logic gates, such as the requirement of several antigens for the activation of CARs (AND GATE) or the generation of inhibitory CARs (iCAR) targeting specifically normal cells, thus the recognition of antigen expressed by healthy cells leads to inhibitory signaling (NOT GATE). Another interesting approach uses hypoxia-regulated CAR-T cells that only express other CARs or molecules under hypoxic conditions.

Alternatively, other groups have aimed at generating smart CAR-T cells which can be controlled by the addition of an inductor, allowing the clinicians to manage the intensity and durability of the therapy reviewed by Tristan-Manzano et al. There are various systems that have been used to kill CAR-T cells if necessary, such as inducible suicide herpes simplex virus tyrosine kinase (HSV-TK) or the iCaspase systems that trigger cell death upon a small molecule administration. In addition, several other systems can control CAR-T activity by controlling the binding of Split-CARs (the CAR binding domain and signaling domains are separated and get together only upon the addition of the inductor) or by controlling the expression of the CAR itself or other molecules (IL12, IFN-β). Several systems have been investigated with this aim, including several TetOn system that uses doxycycline as inductor, the RheoSwich that uses veledimex and the Gene Switch system that use synthetic steroid mifepristone (MFP).

## CAR-T cells for solid tumors

Efforts are ongoing to utilize the CAR-T cell strategy for the treatment of solid tumors, however, the clinical efficacy for these types of tumors is limited as compared to hematological malignancies, due to intrinsic mechanisms of cancer cells leading to their escape from immune responses. The principal factors limiting the clinical development of CAR-T cells are the immunosuppressive tumor microenvironment and the inability of T cells to penetrate the tumor-associated vasculature and the extracellular matrix. Rodriguez-Garcia et al. and Kozani et al., have reviewed the possible strategies to release the brakes of CAR-T cells and implement their efficacy in the targeting of solid tumors.

Examples of these approaches are the manipulation of the TME by either the combination of CAR-T cell infusion with monoclonal antibodies targeting regulatory immune cells or immune checkpoint molecules, the expression of cytokines or chemokine ligands by the CAR-T cells or by modifying the T lymphocytes to be resistant to immune suppression. CAR-T cells targeting stromal cells can also be employed to overcome the inhibitory effect of TME [25]. This knowledge can also be applied to improve CAR-T cell potency. Pavlovic et al. reviewed how CAR-T cells can be re-engineered using genome editing tools to make them resistant to the TME environment or to become more resistant to exhaustion.

The combination of CAR-based cell therapy with chemotherapy or antiangiogenic agents can facilitate the migration of T cells to the tumor milieu. Additionally, the addition of either cancer vaccines or photothermal therapy with cell therapy have been explored to engage the antigen presenting cells and augment the CAR-T cell proliferation, multifunctionality and anti-tumor activity or to recruit bystander immune cells through antigen spreading Rodriguez-Garcia et al., Kozani et al.]. Along this line, the lack or a low number of tumor-specific antigens in solid tumors that are not expressed by normal tissues to prevent the risk of “off-target’ reactivity and toxicities has limited the number of clinical trials of CAR-T cells in solid tumors. The high grade of tumor heterogeneity and the loss of antigen along with tumor progression can also limit the clinical efficacy of CAR-T cells targeting a single antigen. In the search for novel tumor-associated antigens, Ponterio et al. investigated the role of glycosylated antigens in redirecting CAR-T cells toward glioblastoma (GBM) and colorectal cancer. Few other reports explored the development of CAR-T cells targeting PIK7, and αvβ6 integrin in lung cancer and cholangiocarcinoma, respectively [Jie et al., Phanthaphol et al.]. Promising results of the ability of CAR-T cells to provide clinical benefit in recurrent solid tumors have been reported for the targeting of EGFR vIII in a patient with aggressive GBM [Durgin et al.]. This case reports the persistence of the infused CAR-T cells in the circulation for 29 months of follow-up with improvement of patient’s survival (36 months) after tumor recurrence. The analysis of the tumor at the post-infusion timeline showed anti-inflammatory adaptations with decreased levels of EGFRvIII and a moderate expression of the programmed cell death protein 1 (PD-1) by the infiltrating T lymphocytes. This evidence suggests that EGFRvIII CAR-T cells might represent a valuable therapeutic therapy for GBM patients with advanced disease upon its optimization, for instance, through the combination with anti-PD-1 antagonistic antibody. The combination of CAR-T cells with PD-1 blockade indeed revealed its efficacy in a mouse model of Her-2^+^ breast cancer [Li et al.].

An additional approach to implement the targeting of tumor cells by CAR-T cells is the combination with bispecific antibodies that can facilitate the redirection and interaction between effectors cells with target cells of both the engineered T cells and possible bystander lymphocytes [Blanco et al.].

## Universal GT-ACTs

Most CAR-T cell approaches use autologous T cells from the same patient to generate the ATMP. However, there are important limitations of this personalized strategy such as the availability, inclusion criteria and cost. The approach of developing allogeneic/”off-the-shelf” CAR-T cells has been pursued by several groups in order to render the manufacturing of these “living drugs” independent of the availability of patients’ derived peripheral blood lymphocytes (PBLs) and shortening the delay that elapses from the patient’s enrollment and the time of infusion of the drug. Morgan et al. and Pavlovic et al. describe how genome editing (GE) can be used to generate “of-the-self” CAR-T cells that can be used for the treatment of patients with type B malignances. They illustrate how GE can be used to eliminate the TCR and the MHC class I and/or MHC class II molecules to prevent GVHD and allograft rejection. However, the knock-out of MHC can result in the recognition and destruction of the modified CAR-T cells by NK cells. Expression of ligands that inhibit NK cell cytotoxicity (HLA E or HLA G) can be implemented to avoid these situations.

Other cell types rather than T cells to generate “off-the-self” GT-ACT products have also been suggested. An overview of this approach has been provided by Guerrouahen et al. The source of allogeneic immune cells, e.g., Natural Killer (NK cells), PBLs, Cord blood, induced pluripotent stem cells (iPSCs), is a critical point in order to lower the risk of allogeneic toxicities and to allow availability of large numbers of cells for the optimal manufacturing CAR-engineered immune cells [34].

NK-CAR cells provide the advantage of rare induction *in vivo* of toxicities, such as cytokine release syndromes or graft-versus-host disease (GvHD). However, challenges in the manipulation of these cells with difficulties in the engineering with exogenous receptors and the sensitivity to apoptosis, warrant the investigations to optimize the manufacturing of clinical-grade CAR-NK cells [Scmidt et al.]. Efforts are undergoing to optimize the isolation, engineering and *ex vivo* expansions of NK cells [Mantesso et al.]. The dual engineering of primary NK cells with CD19 CAR and chemokine receptor (CXCR4) equipped the engineered immune cells with high efficiency in migrating to the bone marrow as compared to conventional CD19-CAR-NK cells, providing a further tool to implement the efficacy of NK cells for cell therapy [Jamali et al.]. Along this line, Huang et al. have identified a robust CRISPR-based genome engineering platform for NK-92 cells leading to the isolation of immune cells endowed with high cell-mediated and antibody-dependent cellular cytotoxic functions.

## Transgeneic TCR-T cells

Endogenous tumor antigens can be targeted by T cells engineered with a TCR that has been isolated from antigen-specific lymphocytes. This approach has shown clinical efficacy in the context of clinical trials for patients with solid tumors. However, the improvement of the generation of anti-tumor T cells is required to endow these cells with potent cytotoxic activity, the ability to migrate to the tumor site, to penetrate the TME and long-term survival and proliferation. T lymphocytes are engineered with antigen-specific TCRs that could be encoded by viral vectors but also by no-viral gene transfer vectors, such as transposons or messenger RNA incorporated into lipid nanoparticles. The advent of gene editing allowed to make a precise insertion of the exogenous TCR gene transfer and the disruption of endogenous genes encoding for α and β chains of the receptors to prevent the TCR mispairing and to augment the expression on the cells of the novel antigen-specific receptors. Gene editing can also be utilized to control the differentiation of T lymphocytes and maintain their early-stage maturation, similar to what has been described above for CAR-T cells.

Moreover, the selection of the target antigen and of tumor-specific TCR is critical for the downstream manufacturing of efficient anti-tumor cell therapy. Neoantigens generated by somatic mutation in tumor cells represent promising tools for the isolation of highly specific” living drug” bearing exogenous TCRs, as they overcome the risk of inducing anergic responses as in the case of self/endogenous antigens. Moreover, the targeting of this type of antigen can prevent the “off-target” toxicities since they are not expressed by normal tissues. Nevertheless, continuous efforts by several groups are ongoing in the search for the “optimal” candidate tumor antigen(s) and to decrease the complexity and lengthy process of the selection of TCR. Of note, soluble T cell redirecting biologics, either TCR or immunoglobulin-based molecules, represent a novel approach to redirect and facilitate T-cell mediated responses toward cells, also providing the advantage of being independent of the availability of autologous patient’s cells. An overview of the topics listed above was provided in this issue by Manfredi et al., Cai et al. and Jones et al. The selection process of TCR for clinical application and targeting preferentially the alpha-fetoprotein on hepatocellular carcinoma cells and not cross-reacting with proteins expressed by normal cells is described by Luo et al. In addition, the TCR proximal signaling and the molecules involved in this process constitute a complex cascade of molecules, in which genomic variations can lead to the development of different diseases including malignancies. The knowledge of the molecular structure, the functions and the relationship of these molecules is relevant to implement the development of TCR gene transfer and engineering for cell therapy [Kent et al.]. The novel strategy to genetically modify tumor-infiltrating lymphocytes (TILs) with retroviral vectors has shown its feasibility to improve the survival machinery of cells that are physiological endowed with anti-tumor properties [Weinstein-Marom et al.].

## Novel approaches

Among novel perspectives of therapeutic approaches for anti-tumor therapies are the reports of *i.* Fereydouni et al. and Fereydouni et al. suggesting the usage of mast cells (MC), sensitized with Her-2/neu IgE in order to polarize their anti-tumor activities and to be utilized for cell-based therapies. *ii.* The engineering of extracellular vesicles, which are secreted by tumor cells, to be used as vaccines or to block the immunosuppressive functions to elicit or implement the anti-tumor immune responses in solid tumors [Yin et al.].

Overall, the Research Topic documents and collects the huge effort ongoing worldwide in the field of GT-ACT, highlighting the progress and the limitations and how the advent of innovative technologies can accelerate the progress toward the development of more precise manufacturing and superior safety for cell-based therapies (**Figure 1** *Panel C summarizes these aspects*).

## Author contributions

FM, AS and CM contributed to the preparation and revision of the text. All the authors agreed with the content of the editorial. All authors contributed to the article and approved the submitted version.

## References

[1] Micklethwaite KP Gowrishankar K Gloss BS Li Z Street JA Moezzi L Mach MA. Investigation of product-derived lymphoma following infusion of piggyBac-modified CD19 chimeric antigen receptor T cells. Blood (2021) 138(16):1391–405. 10.1182/blood.2021010858 PMC8532197 33974080

